# Exploration of the Activation Mechanism of the Epigenetic Regulator MLL3: A QM/MM Study

**DOI:** 10.3390/biom11071051

**Published:** 2021-07-17

**Authors:** Sebastián Miranda-Rojas, Kevin Blanco-Esperguez, Iñaki Tuñón, Johannes Kästner, Fernando Mendizábal

**Affiliations:** 1 Departamento de Ciencias Químicas, Facultad de Ciencias Exactas, Universidad Andres Bello, República 275, Santiago 8370146, Chile; k.blancoesperguez@uandresbello.edu; 2 Departamento de Química Física, Universidad de Valencia, 46100 Burjasot, Spain; ignacio.tunon@uv.es; 3 Institut für Theoretische Chemie, Universität Stuttgart, Pfaffenwaldring 55, 70569 Stuttgart, Germany; kaestner@theochem.uni-stuttgart.de; 4 Departamento de Química, Facultad de Ciencias, Universidad de Chile, P.O. Box 653, Las Palmeras 3425, Ñuñoa, Santiago 7800003, Chile; hagua@uchile.cl

**Keywords:** cancer, protein regulation, enzyme catalysis, methyltransferase, DFT

## Abstract

The mixed lineage leukemia 3 or MLL3 is the enzyme in charge of the writing of an epigenetic mark through the methylation of lysine 4 from the N-terminal domain of histone 3 and its deregulation has been related to several cancer lines. An interesting feature of this enzyme comes from its regulation mechanism, which involves its binding to an activating dimer before it can be catalytically functional. Once the trimer is formed, the reaction mechanism proceeds through the deprotonation of the lysine followed by the methyl-transfer reaction. Here we present a detailed exploration of the activation mechanism through a QM/MM approach focusing on both steps of the reaction, aiming to provide new insights into the deprotonation process and the role of the catalytic machinery in the methyl-transfer reaction. Our finding suggests that the source of the activation mechanism comes from conformational restriction mediated by the formation of a network of salt-bridges between MLL3 and one of the activating subunits, which restricts and stabilizes the positioning of several residues relevant for the catalysis. New insights into the deprotonation mechanism of lysine are provided, identifying a valine residue as crucial in the positioning of the water molecule in charge of the process. Finally, a tyrosine residue was found to assist the methyl transfer from SAM to the target lysine.

## 1. Introduction

Epigenetic writing such as the methylation of the histone tail is an emerging focus of research because of its involvement in important post-translational processes. Its deregulation has been related to aberrant gene regulation leading to several diseases such as cancer [1]. Normally, methyltransferases target a lysine or an arginine residue from the N-terminal domain from a histone protein. These residues can be mono-, di- and trimethylated with the epigenetic outcome depending on the product multiplicity. The mixed lineage leukemia (MLL) family of proteins corresponds to a set of non-redundant methyltransferases found in humans, from which the deregulation of MLL3 functioning has been detected in several types of tumors, such as breast cancer, gastric cancer, medulloblastoma, non-Hodgkin lymphoma, among several others [2,3,4,5,6,7,8]. MLL3 is a mono-methyltransferase that targets lysine 4 (Lys4) from histone 3 (H3K4), an epigenetic mark that has been related to enhancer elements involved in the activation of tumor suppressor genes, thus its inactivation affects the protective mechanism against tumor development [9,10].

**Scheme 1:**
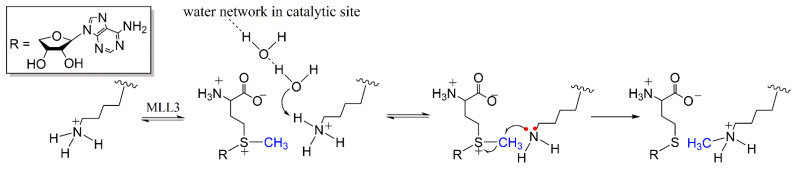
Proposed reaction mechanism for the deprotonation step and methylation process of the lysine residue by MLL3 (adapted from ref. [17]).

**Figure 1:**
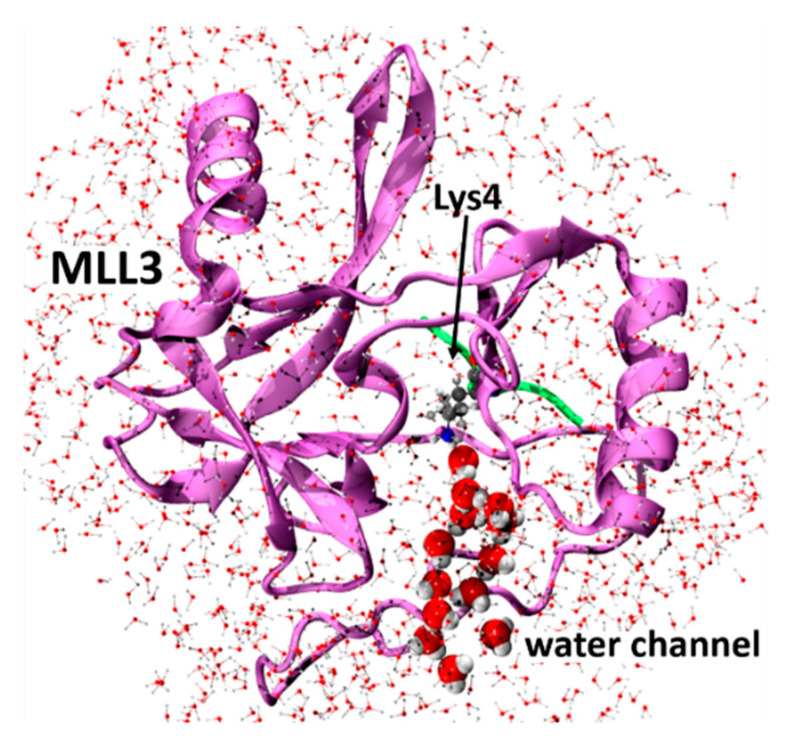
Graphical representation of the water channel connecting Lys-ε-NH_3_^+^ with the outside of the MLL3 enzyme. (purple―MLL3 subunit; green―peptide substrate; gray―carbon; red―oxygen; blue―nitrogen; white―hydrogen).

The reaction process shown in Scheme 1 involves the initial binding of S-adenosyl methionine (SAM) as a cofactor and source of the methyl group to be transferred, which stabilizes the substrate binding site [11]. Then, the Lys4 binds to the enzyme at an adjacent site to the cofactor. An important subsequent step of the reaction involves the deprotonation of the amine group of substrate lysine (Lys4-ε-NH_3_^+^ for MLL3 and Lys-ε-NH_3_^+^ in general), which in solution has a pKa > 10, meaning that it is mostly protonated at pH 8, pH at which the activity assays of MLL3 were performed [11]. Posterior to the deprotonation, Lys4 is attacked by the methyl group. In this sense, the deprotonation mechanism is still a subject of debate. Initially, it was proposed that a tyrosine from the catalytic site acted as a general base deprotonating the Lys-ε-NH_3_^+^ [12,13,14]. However, based on the increasing crystallographic evidence, particularly from the protein lysine methyltransferase (PKMT) of Rubisco pointed out that the suggested tyrosine was incapable of deprotonating the Lys-ε-NH_3_^+^, alternatively suggesting a mechanism based on deprotonation by bulk solvent [15]. This mechanism was also explored through computational methods by using an hydroxyl molecule to simulate a basic environment inside the water channel suggested to be responsible of the deprotonation; resulting in a barrierless process [16]. However, at the optimal pH at which MLL3 was shown to be catalytically efficient (7.8) [11], this mechanism is unlikely to drive the reaction because of the free energy cost associated to the production of a hydroxyl anion. In this context, a thorough experimental study focused on the kinetics and mechanistic insights involved in both the deprotonation and the methyl-transfer process on the PRC2 enzyme was carried out by Kipp et al. [17] In this work, they found that inside the enzyme environment, the pKa of Lys-ε-NH_3_^+^ is reduced from the classically known value of ~10.5 to ~7–8, in line with computational results where a reduction to ~8.2 was found and attributed to the electrostatic repulsion between Lys-ε-NH_3_^+^ and SAM [18]. From solvent kinetic isotope effect (sKIE) experiments, it was suggested that the environment drives the deprotonation mechanism, specifically the water molecules present in a water channel next to Lys-ε-NH_3_^+^. It is proposed that the binding of the substrate could stabilize this water channel. However, even more remarkable was that the experimental data suggested that the deprotonation step is at least partially rate-limiting exposing the relevance of this step on the reaction mechanism, which was previously only focused on the methyl-transfer process. It has been shown that other methyltransferases such as SET9/7, LSMT and vSET also exhibit the required water channel necessary for the water driven deprotonation mechanism [18]. The accessibility and stabilization of the water channel seem to control the methylation process reaction and multiplicity. In this regard, visual inspection of the crystal structure of MLL3 shows a water channel that goes from the catalytic site to the outside of this enzyme, suggesting a similar mechanism for the deprotonation step (see Figure 1).

On the other hand, an interesting feature of MLL3 is that despite containing the SET domain known to provide the catalytic machinery to perform the methyl-transfer process, it is inactive by itself. To be activated, it needs to bind with a protein dimer formed by ASH2L and RBBP5, dimer that has to be formed before its interaction with MLL3 [11]. Thereby, the formation of the trimer between MLL3-ASH2L-RBBP5, from now on named M3RA, the active form of the enzyme, acts as a regulation mechanism for the activity of the enzyme. The mechanism by which ASH2L and RBBP5 activate the enzyme is the main focus of the present research.

Along the process of understanding the regulation mechanism, Li et al. [11] found that the binding with the dimer increased the affinity of the SAM cofactor for the enzyme from K_D_ = 78 ± 8 μM for MLL3 to K_D_ = 6.9 ± 0.7 μM for M3RA, as determined by isothermal titration calorimetry (ITC) measurements. Also, through fluorescence polarization assay, they showed that there is also an increase of H3 peptide binding affinity. To rationalize these two outcomes, they carried out a normal mode analysis between the trimer and the monomer. These results suggested that the trimer presents higher conformation stability reflected by lower dynamic motions than the monomer. Also, they performed quantum mechanics/molecular mechanics (QM/MM) calculations obtaining an energy barrier of ca. 25 kcal/mol and ca. 30 kcal/mol for the trimer and monomer, respectively, with a decrease in the energy barrier close to 5 kcal/mol. Finally, they proposed that the binding of the RBBP5–ASH2L dimer to MLL3 (SET domain) induced conformational constraints to this subunit that allowed to stabilize conformations able to favor cofactor binding and substrate recognition.

Here we focused on providing a closer look at the molecular basis by which the binding of the RBBP5-ASH2L dimer to MLL3 regulates its catalytic site and thus, the activity of the enzyme. In this context, we first explored the mechanism for the deprotonation process and the feasibility of the bulk solvent deprotonation mechanism; followed by the study of the methyl-transfer reaction. For this purpose, we present a QM/MM study of an exhaustive set of conformers obtained from molecular dynamics simulations as an approach to rationalize the regulation mechanism. By exploring the reaction mechanism through QM/MM models, it was possible to reveal with high accuracy the microenvironment mechanisms involved in the regulation process that are the focus of this study. This, in contrast to the analysis of global conformational changes that only provides a macro-picture of the regulation mechanism, which is not always useful for the design of new inhibitors for the enzyme. Also, the detailed analysis of the deprotonation mechanism opens a new branch in the design of potential inhibitors at this step of the reaction. The insights obtained sheds new light on our understanding of the regulation mechanism of similar methyltransferase enzymes.

## 2. Materials and Methods

### 2.1. Model Preparation and MD Simulations 

The crystal structure of the human trimeric functional system MLL3-ASH2L-RBBP5 in complex with an H3 segment as a peptide substrate was obtained from the crystal structure deposited in the Protein Data Bank (PDB) with entry 5F6K [11]. The protonation states of the titratable residues were determined using PropKa [19], considering a pH of 7.8 according to the pH used in the activity assays from Li et al. [11]. Then, VMD (v1.9.1) [20] was used to protonate and solvate the enzyme in a rectangular box of TIP3P water molecules. Ions were added to reach a concentration of 0.05 mol/L and to ensure charge neutrality of the system. MD simulations were carried out using periodic boundary conditions as implemented in the NAMD code version 2.9 [21], and the CHARMM22 force field [22,23]. The force field parameters for the SAM cofactor were obtained from a previously reported set of parameters [24]. The time step was set to 2 fs and the Langevin piston Nosé-Hoover method [25] was used to keep the temperature and pressure at 300 K and 1 atm. The bonds involving hydrogen atoms were restrained by the SHAKE algorithm as implemented in NAMD [26]. The equilibration protocol was performed in four stages before starting the production dynamics. First, the water molecules are allowed to relax while the protein was kept fixed through 2000 conjugate gradient steps and 0.1 ns of MD simulation. This was done to accommodate the water molecules located next to the cavities around the surface of the protein. Next, three subsequent MD calculations of 0.1 ns each were run with a force constant restraint in the protein of 5.0, 2.0 and 0.1 kcal/mol·Å^2^. For the conformational sampling of the active and inactive systems, MD simulations were prepared for the trimer formed by the MLL3-RBBP5-ASH2L subunits (M3RA) and for the monomer defined as MLL3; being the latter the model for the inactive system. In addition, to study the two steps of the reaction mechanism, namely the deprotonation and the methyl-transfer, for both M3RA and MLL3 were prepared MD simulations with the substrate lysine in its protonated (Lys4-H^+^) and deprotonated state (Lys4). Finally, a 50 ns MD calculation without restraint was performed and defined as our production trajectory for the systems (M3RA and MLL3) with the deprotonated Lys4. Meanwhile, only 20 ns MD simulations were possible for the protonated substrate because after that timeframe, the cofactor tends to increase its distance with Lys4-H^+^ due to the high electrostatic repulsion, and even in one parallel simulation the cofactor dissociated from the binding site.

### 2.2. QM/MM Calculations

As the main goal of this work is to shed light on the activation mechanism by which the binding of the dimer (RBBP5-ASH2L) to MLL3 activates the enzymatic activity, it is necessary to use an approach capable of incorporating the total structural differences between the active and inactive system while studying the chemical process. In this sense, the QM/MM approach offers the most realistic representation of the system as required, allowing to explore the reaction mechanism in the active and inactive state of the enzyme. QM/MM offers the best balance between accuracy and efficiency when dealing with problems involving large, reactive systems. The initial structures were obtained from the MD simulations, from which we selected a series of snapshots to start the QM/MM geometry optimizations. The snapshot selection for the methyl-transfer reaction was straightforward taking one conformation every 2 ns until 30 ns of simulation were reached; after which one conformation was selected every 4 ns from 30 to 50 ns having a total of 20 conformations. The selection was carried out considering the shortest SAM-C···N-Lys4 distance within each sampling window and the largest S···C···N angle, ensuring to have a separation of at least 500 ps between two conformations taken from adjacent sampling windows. Considering that we expected a reaction energy barrier dependent on the initial conformation and to capture the structural features that characterize the active system, this strategy was considered a better approach than taking a random snapshot at the end of every 2 ns window. This was implemented for MR3A and MLL3. The snapshot selection for the deprotonation process was a challenging process. Every 2 ns window it was selected a conformation where a set of water molecules were forming a structural chain from Lys-H^+^ to the outside of the enzyme. Due to the complexity of considering several simultaneous interactions between the water molecules, visual inspection was used to select the conformation considered best suited for a proton transfer process. In general, the QM/MM model was prepared from the snapshot by considering the full enzyme and 5 Å of water molecules surrounding the protein, being this defined as the QM/MM system. Then, we have the active region, which is the region allowed to move and relax during the optimization process, and therefore, everything outside of the active region is kept fixed during the geometry optimizations. For the models with Lys4, the active region was defined as all residues and water molecules within 8 Å from the sulphur of the SAM cofactor. For the models with Lys4-H^+^ the active region was defined as all residues and water molecules within 12 Å from the nitrogen of the substrate. This larger active region for the modeling of the deprotonation process was necessary to incorporate the complete water network connecting the Lys4-H^+^ at the inside of the catalytic site with the outside of the protein, where the proton should be finally transferred. It should be stated that with smaller active regions, it was not possible to locate a converged TS for the deprotonations process. This was attributed to the fact that in order to achieved the proton transfer through the water network, the whole set of water molecules needed be able to freely relax during the optimization process to adopt the best suited orientation for the reaction.

**Figure 2:**
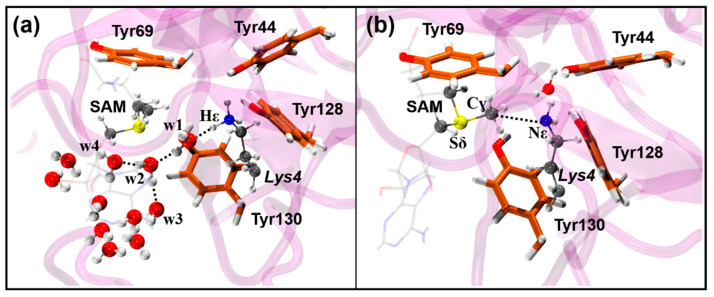
QM region used to study the (**a**) deprotonation and (**b**) methyl-transfer process. The respective reaction coordinates used to explore the transition states for each case are depicted. As the deprotonation reaction was found to be a highly concerted step occurring between the water molecules forming the water channel, there are several possible pathways denoted by the two routes shown in (**a**) towards w3 or w4. (enzyme’s carbon―orange; substrate and cofactor carbon―gray; red―oxygen; blue―nitrogen; white―hydrogen).

The QM region to study both stages of the reaction mechanism is graphically represented in Figure 2. The correlation between the residues numbering used in this article and the one used in the article from Li et al. [11], is explained in Table S1. The QM region used for the methyl-transfer process involved part of the SAM cofactor, the substrate (Lys4), Tyr44, Tyr69, Tyr128, Tyr130 and a water molecule interacting with Tyr128, hereinafter defined as WY_128_, with a total of 93 atoms. For the deprotonation process, in addition to the above defined fragments, every water molecule involved in the chain connecting Lys4-H^+^ with the outside of the enzyme was included in the QM region, without including any water molecule from the outer limit of the QM/MM system, as these are kept frozen during the optimizations. The number of molecules forming the water network along the simulations was not constant, and varied between 9 and 15 water molecules, which were included in the QM region. According to this, the number of atoms of the QM regions ranged between 118 and 136 atoms, with 9 and 15 water molecules; respectively. All the residues included in the QM region are truncated between the Cα and Cβ, while for SAM the sulphur and methyl groups directly bonded to it were considered as part of the QM region.

All geometry optimizations were carried out at the DFT level of theory using B3LYP as the density functional [27,28], which has proven to provide accurate results for other methyltransferases [29,30,31]. For the basis set, the all-electron def2-SVP was used for the optimizations [32], followed by an energy correction by using the def2-TZVPP basis set [33]. All DFT calculations include the DFT-D3 method (with the Becke-Johnson damping function) [34,35,36].

The transition state search for the Lys4-H^+^ deprotonation process involved a reaction coordinate defined by the difference of three distances calculated as follows [O(W_1_)···H(W_1_) − Hε(Lys4)···O(W_1_) − H(W_1_)···O(W_2_)]. This coordinate allows the description of the proton transfer throughout the water channel. Meanwhile, for the transition state search of the methyl transfer process, a reaction coordinate defined by the difference of distances [Nε(Lys4) ··Cγ(SAM) − Sδ(SAM)···Cγ(SAM)] as shown in Figure 2 was used, as suggested by Bruice et al. [16], as a mean to avoid the overestimation of reaction barriers. For both cases, meaning the deprotonation and methyl transfer reaction, once a maximum was found on the potential energy surface, transition state (TS) optimization was carried out using the dimer method [37]. All QM/MM calculations were performed using the ChemShell suite [38], coupled with Turbomole v7.3 [39] to perform the electronic structure calculations involving the QM region. In contrast, the calculations of the MM region were performed with the DL-POLY program as implemented in the Chemshell suite.

To provide a qualitative picture of the contribution and topology of the non-covalent interactions taking place inside the catalytic site, we calculated the non-covalent interaction index (NCI) [40,41]. From this analysis, it is possible to obtain a representation in real space of the non-covalent interactions between the reacting parts, namely SAM and Lys4 with the enzymatic environment. This allows identifying the stabilizing contributions coming from the dispersion interactions provided by the enzymatic environment to the reaction process. Also, it permits the distinction between attractive and repulsive interactions. For interpretative purposes, the regions of the surface colored in blue denote strong stabilizing interactions, green indicates weak interactions usually associated with van der Waals interactions, and the colored in red are indicative of repulsive interactions. Finally, we carried out the natural population analysis (NPA) [42] to analyze the electronic charge redistribution taking place along the reaction process. For clarity, we defined reactant state, transition state and product state as RS, TS and PS; respectively. In addition, to simplify the analysis of the several snapshots here studied, for the deprotonation process, each snapshot that converged to a proper TS was sequentially numbered according to the nomenclature T(M)h-conf-N, with N being the identifier and T (or M) indicates the trimer (or monomer). For the methyl-transfer process, as the sampling was straightforward, we used T(M)-conf-N, where N denotes the window of sample from which it was taken. For example, T-conf-1 corresponds to a snapshot taken from the first 2 ns of simulation of the trimer.

## 3. Results and Discussion

### 3.1. Activation of the Deprotonation Process

**Figure 3:**
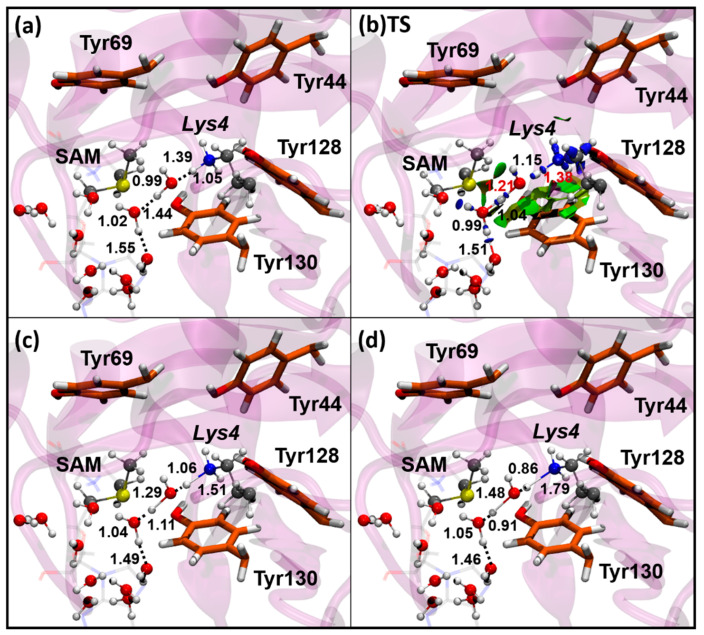
Sequential representation of the evolution through the normal mode associated to the reaction coordinate for the deprotonation process in M3RA. For clarity, only the QM atoms are shown. The evolution of the Lys4(N)-H^+^ bond through the reaction normal mode is represented sequentially from (**a**–**d**). The concerted nature of the process is revealed involving three water molecules in the process. The second structure denoted as (**b**) TS corresponds to the geometry at the transition state of the reaction, where the surface corresponding to the DORI analysis is also included, which reveals the role of Tyr130 in the proper alignment of Lys4-H^+^ and the water channel. (enzyme’s carbon―orange; substrate and cofactor carbon―gray; red―oxygen; blue―nitrogen; white―hydrogen and the isovalue used as cutoff for the surface was defined at 0.5).

The study of the deprotonation process was highly complex in terms of finding suitable TS structures due to the high number of degrees of freedom involved in the process. The first finding was that the reaction is a concerted process where the proton from Lys4-H^+^ is removed by a water molecule (defined as W_1_) and simultaneously transferred sequentially through the water chain outside of the enzyme, involving at least three water molecules as depicted in Figure 3. To properly model the process, a reaction coordinate involving several interaction distances was used as defined in the methodology section. Any attempt to remove the proton by only using the elongation of the Nε―H bond or the difference of distances between the Nε―H^+^ bond and the H^+^―W_1_ interaction led to reaction profiles of monotonically increasing energy, always surpassing the 30 kcal/mol without reaching a maximum.

On the other hand, the second limitation was the simulation window used for sampling, which was limited to 20 ns. After this time, the cofactor tends to slightly dissociate from its binding site generating what could be interpreted as artificial conformations due to the electrostatic repulsion exerted by the Lys4-H^+^ substrate. According to this, it is expected that the deprotonation step takes places immediately after the substrate binding. From the ten conformers sampled from the M3RA simulations to search suitable TSs for the deprotonation, only four derived in the convergence of TSs for the deprotonation reaction. The calculated barrier heights (∆E^≠^) for the deprotonation reaction of M3RA ranged between 10.9 and 16.5 kcal/mol.

From the normal mode of the reaction coordinate of Th-conf3 depicted in Figure 3, it is possible to expose the concerted nature of the process, involving several water molecules, probably necessary for a fast proton removal from the surroundings of the SAM cofactor. The difficulties in finding appropriate conformers for the TS of the deprotonation process also reveals the reduced accessibility of this conformation even for the active system (M3RA), as a consequence of the highly complex nature in terms of degrees of freedom. The values of ∆E^≠^ for MLL3 were within 19.5 and 33.5 kcal/mol, which resulted in a marked increase of ∆E^≠^ compared to M3RA. To understand the source of this increase, we analyzed the interactions between the reacting parts and the catalytic site finding that the most important interactions were Tyr44-Lys4-H^+^, Tyr128-Lys4-H^+^, Val68-W_1_ and Tyr69-SAM as listed and defined in Table 1. The rest of the interactions did not show major changes between the active and inactive systems. From the mean values, it is possible to observe that the interactions between the tyrosines and lysine showed, in general, shorter distances for the active system, denoting the need to keep the protonated group into a more restricted position. For the particular case of Th-conf-2, it showed larger Tyr128-Lys4-H^+^ distances, but this was compensated by a water molecule that was positioned in between the two molecules (Tyr128 and Lys4-H^+^) fixing the position of lysine. An interesting feature was the role of Val68 in the deprotonation process, which directly interacts with W_1_ being the residue in charge of the proper positioning of the water molecule. The mean values at the RS were 2.63 and 3.22 Å for M3RA and MLL3, respectively; which denotes that shorter distances between Val68 and W_1_ lead to lower values of ∆E^≠^ for the deprotonation process. The relevance of this interaction is supported by the fact that Mh-conf2 showed the lowest ∆E^≠^ and the shortest Val68-W_1_ interaction distance. According to these results, a suitable proposal could be that the binding of the RBBP5-ASH2L dimer regulates this step of the reaction by the proper positioning of the Tyr44, Tyr128 and Val68 residues in the catalytic site. The longer interaction distances observed for Tyr69-OH with the carboxylate from SAM MLL3 also denotes the lack of an appropriate binding of the cofactor in the inactive system. It should be considered that the SAM cofactor is mainly anchored by its two charged groups, namely the carboxylic acid and the protonated amine. Thus the loss of one of these anchoring points on MLL3 probably leads to a lower affinity for the binding site as experimentally observed [11], point that will gain relevance during the analysis of the methyl-transfer process. To define the non-covalent interactions taking place in the catalytic site during deprotonation process, we used the Density Overlap Regions Indicator (DORI) [43], which incorporated the NCI index together with the covalent component of the interactions. An interesting feature revealed by the NCI index shown in Figure 3 was the involvement of Tyr130 in the proper alignment between the Lys4-H^+^ and the water molecules, where acts as structural support to keep the conformational requirements for the reaction through dispersion interactions.

### 3.2. Methyl-Transfer Reaction Mechanism

The next step on the enzymatic mechanism after the deprotonation of Lys4-H^+^ corresponds to the methyl-transfer reaction. For this, we proceeded by studying the process starting with conformations obtained from the MD simulations of the enzyme with the deprotonated Lys4, assuming that the deprotonation process already took place, and from there, we proceeded with the QM/MM calculations. We also analyzed the methyl transfer step before the proton leaves the water channel but we found that the energy barriers were considerably higher than when the excess proton has been completely released to the bulk (see Supplementary Materials, Section S1).

#### 3.2.1. Methyl-Transfer Energy Barrier

To analyze the calculated energy barriers (∆E^≠^) first, it is necessary to establish a baseline to separate those systems that will be considered as catalytically active from those that will be defined as catalytically inefficient. According to the experimental data listed in Table S2, most of the experimentally-derived activation free energy barriers are close to 20 kcal/mol or below. Therefore, it is reasonable to define the system as catalytically inefficient if it shows a decrease of four orders of magnitude of the rate constant (k_cat_), this is if the activation free energy barrier increases up to 25 kcal/mol. This value has been here selected to define the baseline separating active and inactive systems. According to this, the calculated ∆E^≠^ for the twenty M3RA systems resulted in four ∆E^≠^ higher than 25 kcal/mol. Even in the active M3RA system, the conformational flexibility found experimentally [11], may lead towards less efficient conformations, but in a lower proportion than the active ones as evidenced by our results. The mean value for ∆E^≠^ considering the twenty conformers listed in Table 2 and Table 3, resulted in a value of 18.9 kcal/mol for M3RA, whereas for MLL3 it was of 30.1 kcal/mol. These magnitudes are in line with our criteria for defining the system as active or inefficient catalytically, where M3RA shows close agreement with the experimental free energy barrier reported for this system (21.3 kcal/mol, see Table S2). Considering that the deprotonation step is at least partially rate-limiting [17], it is expected to present a ∆E^≠^ somewhat smaller than for the methyl transfer step. Our results show that the ∆E^≠^ for the deprotonation reaction catalyzed by M3RA is 4.4 kcal/mol smaller than the ∆E^≠^ for the methyl-transfer step. Meanwhile, for MLL3 the difference is only of 1.9 kcal/mol. The difference between the reaction barrier for the deprotonation and the methyl-transfer reaction for both systems partially evidence the experimental data, thereby supporting the observation that the deprotonation step is at least partially rate-limiting [17]. To this date, this is the first model capable of rationalizing this experimental observation.

#### 3.2.2. Role of the Donor-Acceptor Distance

To understand the energetic behavior for the methyl-transfer reaction, we first analyzed the main geometric parameters involving the atoms that are part of the reaction axis, meaning Sδ(SAM), Cγ(SAM) and Nε(Lys4). The selected geometric parameters, namely the S···C, C···N distances and S···C···N angle are listed in Table 2 and Table 3 for M3RA and MLL3, respectively. The mean values of the C···N distances for M3RA and MLL3 at the RS were 3.19 and 3.60 Å, respectively; exposing the longer C···N distance as a general trend when comparing the active (M3RA) versus the inactive (MLL3) enzyme. Regarding the TS, the mean values of the S-C distance show a longer bond for MLL3 (2.38 Å) than for M3RA (2.30 Å), which suggests that the monomer needs to stretch the S-C bond to a larger extent compared to the case of M3RA. This could be a consequence of the longer C···N distance and be partially responsible for the higher ∆E^≠^ observed in the majority of the MLL3 conformers.

The methyl-transfer process is a S_N_2 reaction in nature, for which the angle between the atoms from the reaction axis attributed to S···C···N should be close to 180° at the TS due to the type of orbital interaction needed for the reaction. Therefore, to minimize the structural work to reach this target angle, it is expected to be as close as possible to 180 at the RS. From the set of conformations, there are systems with angles ranging from 108.2° (T-conf-17) to 170.9° (T-conf12) at the RS for M3RA, which according to their ∆E^≠^ represent an inactive and active system; respectively. Meanwhile, the angle values for MLL3 were in general lower than the observed for M3RA, which at the RS showed an average value of 151.0° compared to the 134.5° obtained for MLL3. This shows a lower and less favorable trend for the S_N_2 attack on the monomer system. Also, at the TS M3RA is able to achieve an angle closer to 180° when compared to MLL3.

**Figure 4:**
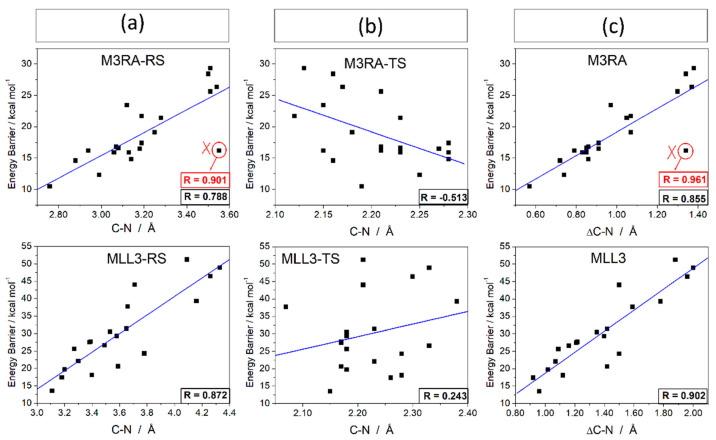
Plot of the distance between the atoms involved in the reaction coordinate for the methyl transfer process, namely the methyl carbon from SAM and the nitrogen from Lys4, versus the energy barrier (∆E^≠^). The correlation factor for the complete data set is in black, and in red is the factor after removing the data in the red circle. (**a**) Reactant state (**b**) Transition state (**c**) Difference between the distances from the reactant and the transition state geometries.

The next step was the exploration of possible relationships between the magnitude of these geometric parameters and the height of the ∆E^≠^. For this purpose, plots of the C···N distances against the ∆E^≠^ were obtained and depicted in Figure 4, while for S···C and S···C···N, these are presented in Figures S1 and S2 in the supporting information. For M3RA, the plot of ∆E^≠^ versus the C···N distance in the RS shows a correlation factor (R) of 0.788, pointing out that longer interaction distances derived into higher ∆E^≠^ as observed for T-conf-13, T-conf-15, T-conf17 and T-conf18, the four conformations with ∆E^≠^ ≥ 25 kcal/mol and C···N interaction distances ≥ 3.50 Å. The only exception was T-conf-9 with a C···N distance of 3.55 Å and ∆E^≠^ of 16.2 kcal/mol, being this the only conformation out of the general trend. If we take this conformation out of the set, the correlation factor (R) increases to 0.901, denoting a clear linear correlation. On the other hand, T-conf2 showed a ∆E^≠^ of 10.5 kcal/mol with the shortest C···N interaction distance of 2.76 Å. In general, the same behavior was observed for MLL3, where the increase in the C···N distance derived in higher ∆E^≠^.

For the TS, there is a high dispersion of the data with no clear trend. Remarkably, the correlation between ∆E^≠^ and the distance traveled by the Met from the RS and the TS given by ΔC···N = [(C···N)_RS_ − (C···N)_TS_]; led towards an increase in the correlation factor (R) to a value of 0.855 and even of 0.961 if we remove T-conf-9. This result reveals that the barrier height linearly depends on the structural work needed for the displacement of the Met group necessary to perform the methylation process. This trend is also observed in the MLL3 monomer, with a correlation factor (R) of 0.902 between ∆E^≠^ and ΔC···N (see Figure 4). Across the series, there is no linear correlation between the S-C-N angle and the ∆E^≠^ in the RS for both M3RA and MLL3 systems, as shown in Figure S1. Nevertheless, the calculated ΔS-C-N from the RS and TS show that the low ∆E^≠^ (active systems) are partially grouped in the region of smaller modifications in the S···C···N angle, in line with the suggested requirement for reduction of the structural work.

The main difference between MLL3 and M3RA comes from the fact that MLL3 presents C···N interaction distances significative longer than those observed for M3RA.

Cases with C···N distances longer than 4.0 Å, such as M-conf6, M-conf7, Mconf-9 and M-conf10, showed ∆E^≠^ values of 48.9, 46.4, 39.3 and 51.2 kcal/mol; respectively. These results point out that over a certain threshold for this interaction distance, the enzyme becomes inactive, suggesting that the enzyme regulation mechanism is related to the modulation of this interaction distance. Interestingly, seven MLL3 (monomer) conformations have energy barriers below 25 kcal/mol, thus considered catalytically active. According to this data, it could be argued that the regulation mechanism is related to a conformational selection in analogy to an allosteric activation mechanism [44], where the active conformation is accessible by the less active system, but the binding of the activator (RBBP5-ASH2L in this case) increases the accessibility and thus the population of the active state. The next step would be to identify the structural moieties from the catalytic site responsible for the longer C···N interaction distances observed for the MLL3 system.

#### 3.2.3. Role of the Enzymatic Environment

**Figure 5:**
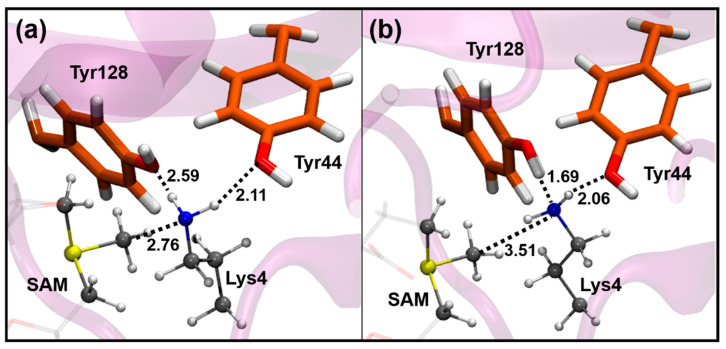
Role of the proper configuration for the interaction between Tyr44 and Tyr128 with Lys4. When both tyrosines act as proton acceptors provide a C···N interaction distance with for (**a**) corresponding to T-conf-2 leads to a ΔE^≠^ of 10.5 kcal/mol, while for (**b**) the T-conf-15 system shows the consequences when Tyr128 acts as a proton donor forming a shorter interaction with Lys4 leading to a ΔE^≠^ of 29.3 kcal/mol.

Concerning the role of the catalytic machinery provided by the environment, for M3RA, it is possible to identify that, in general, to have a catalytically active system the residues Tyr44 and Tyr128 must act as proton acceptors of the NH_2_ group from Lys4 as shown in Figure 5a. Otherwise, if Nε(Lys4) acts as a proton acceptor of one of the tyrosine hydroxyl groups, as depicted in Figure 5b, the resulting configurations display larger C···N distances, thereby increasing ∆E^≠^. For example, T-conf-13 and T-conf-15 have Tyr128 acting as a proton donor to Nε(Lys4), presenting Tyr128-OH···N(ε)-Lys4 distances of 1.66 and 1.69 Å, respectively; which in both cases leaded to a C···N interaction distance of 3.51 Å and ∆E^≠^ of 25.6 and 29.3 kcal/mol, respectively. Thereby, according to what was defined above, these systems should be considered as catalytically inefficient. The results suggest that these two tyrosines play a crucial role in the regulation of the C···N interaction distance. Similar behavior was observed for MLL3, reinforcing this proposal. M-conf9, M-conf-10 and M-conf-12 configurations showed no interaction between Lys4 and Tyr44 neither at RS or TS, but displayed a hydrogen bond interaction between Tyr128···Lys4, thereby leading to larger C···N distances and consequently to high ∆E^≠^ values. As shown in Table 4, for MLL3 the Tyr44···Lys4 interaction distances are in general larger than in M3RA, showing that the conformation of Tyr44 is the most affected by the absence of the activator dimer (RBBP5-ASH2L). Therefore, our results pinpoint this residue in particular as relevant in the regulation mechanism. For M-conf6 and M-conf7, there is an inversion of the Tyr128-Lys4 interaction, where Lys4 is now acting as the proton acceptor leading to short interaction distances responsible for large C···N distances and finally, high ∆E^≠^.

The mean values from Table 4 indicate that the Tyr44···Lys4 interaction for M3RA and MLL3 suffered an increase after going from the RS to the TS from 2.30 to 2.43 Å for M3RA and from 2.95 to 3.22 Å for MLL3. The larger elongation in MLL3 is to allow the amine group from Lys4 to approach the attacking Met group, because it is at a longer distance than in M3RA. The same behavior is observed for Tyr128 in MLL3. At this point, the results suggest that without the binding of the activator dimer (RBBP5-ASH2L) to MLL3, the tyrosines tend to partially lose their conformational stability necessary to provide the appropriated C···N distance. However, as we will point out in the last section involving the analysis of the MD simulations, the longer C···N distance is also a consequence of the proper positioning of the SAM cofactor, which in absence of the activator dimer loses one anchoring point, thus being unable to locate the Met group at a shorter distance from Lys4.

**Figure 6:**
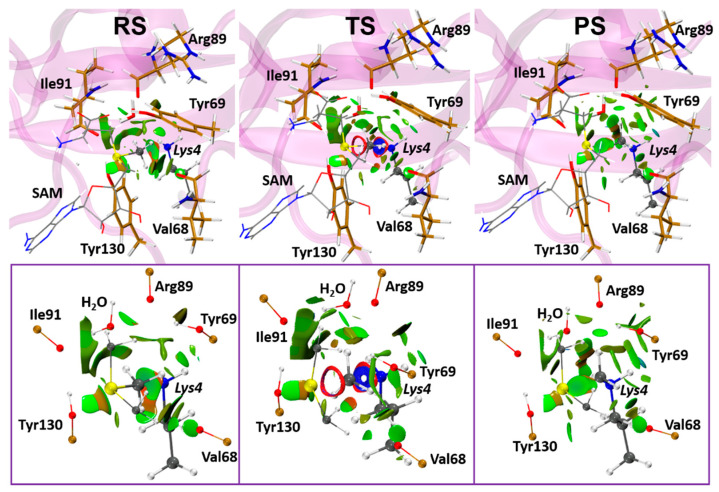
Graphic representation in real space of the non-covalent interactions taking place between the methyl group and the catalytic site along the reaction process. Below there is a simplified picture of the interactions where only the C=O moiety from backbone and C-O-H par of Tyr moieties are shown for the residues involved in this oxy-anionic ring (enzyme’s carbon―orange; substrate and cofactor carbon―gray; red―oxygen; blue―nitrogen; white―hydrogen and the isovalue used as cutoff for the surface was defined at 0.5).

At the region connecting the binding sites of SAM and the substrate, there is a group of residues shown in Figure 6 that form a sort of an oxy-anionic ring surrounding the channel through which the Met group travels from SAM to the substrate (Lys4). Its role could be attributed to the stabilization of the positively charged methyl cation during its transfer, thus stabilizing the TS and working as one of the driving forces of the reaction. The residues involved are Val68, Arg89 and Ile91, which contribute with their backbone carbonyl oxygens to this oxy-anionic ring structural feature; Tyr130 also contributes with the hydroxyl group together with the W_Y128_ that closely interacts with Tyr128 defined as part of the QM region. The interaction distances between Met and Val68, Tyr130 and W_Y128_ tend to decrease in most cases when going from the RS to the TS for both M3RA and MLL3. This points out that these interactions may get stronger at the TS having a role in its stabilization as also discussed in a previous work about the mechanism of a glycine methyltransferase [45].

From the mean values, it is possible to establish some trends for the interaction distances between Met and the anionic ring of M3RA and MLL3. All distances are between the carbonyl oxygen from the backbone of each residue and the closest hydrogen atom from the Met group. In terms of the actual distances, Val68 showed a decrease from 3.14 (RS) to 2.90 Å (TS) for M3RA, while for MLL3, it decreases from 2.64 (RS) to 2.53 Å (TS). On the other hand, Tyr130 showed a decrease from 3.11 (RS) to 2.93 Å (TS) for M3RA, and for MLL3, it decreases from 3.60 (RS) to 2.78 Å (TS); also, the analysis of the individual conformations show that this interaction is not present on several of the MLL3 systems. For the case of Arg89, M3RA keeps close to the same distance going from 3.20 (RS) to 3.22 Å (TS), whereas for MLL3 it goes from 2.85 (RS) to 3.30 Å (TS) showing a significant increase for the latter. The interaction between W_Y128_ and Met was similar for M3RA and MLL3.

Except for Val68, the rest of the interactions between Met and the residues from the anionic ring showed larger variations between RS and TS for MLL3 than M3RA. This exposes that the conformation of the catalytic site of M3RA is best suited to accommodate the TS when compared to MLL3, which has to suffer more extensive structural modifications than M3RA to adapt its structure. In addition to the expected stabilizing effect of the anionic ring, the interactions formed with the Met group help to keep this attacking group centered in the reaction trajectory without deviations providing an outer-shell stabilizing field. The use of the non-covalent interaction index (NCI) allowed to establish the nature of these interactions and provide a qualitative measure of their strength. For this purpose, it was selected the system with the lowest ∆E^≠^ from M3RA (T-conf-2). The wavefunction of the QM region was recalculated by incorporating all the residues considered as part of the anionic ring above defined. The results depicted in Figure 6 reveal the dispersive nature of these interactions exposing in real space the intricated interaction network formed between the Met group and the anionic ring. In this sense, the interactions can be defined as weak hydrogen bonds mainly between the hydrogens from Met with oxygens from the carbonyl moiety of the backbone of the residues above mentioned and the W_Y128_ molecule interacting with Met. The green color of the surface between each of these interacting atoms is indicative of weak but attractive dispersive interactions. Despite that the nature of these interactions may have an electrostatic contribution, it can be argued that the dispersive interactions are the responsible for acting as a conducting force allowing the reaction to progress to the TS geometry. The interactions here described originate from the dipole-dipole interaction coming from the polarized C(δ^−^)-H(δ^+^) bond from the Met group and the permanent dipole from the C(δ^+^)=O(δ^−^) bond from the backbone. The advantage of this type of interactions is their dependence on the directionality as dipolar moments have a specific orientation; therefore, if the TS geometry allows to adopt a better suited orientation for these interactions, this will act as a driving force towards the TS formation. This is the case for the system here studied, as is actually evidenced by the shortening of the distances observed particularly for M3RA. An interesting additional feature that arises from this analysis is the strong attractive interaction at the TS depicted by the blue ring surface between the Met and the Nε from Lys4, while with Sδ is only repulsive, which may aid to promote the reaction towards the products and partially explain the irreversible nature of this step [17].

Regarding the effect of the activator dimer in the cofactor binding stabilization, we found that the SAM-Tyr69 interaction works as an indicator of the structural stability of this cofactor conformation inside the binding site. For M3RA, this interaction distance remains close to the mean value of 1.57 Å at both RS and TS independent of the point of the simulation from which the conformation was taken. For MLL3, on the other hand, this interaction is lost in several conformations starting from the 18 ns of simulation, reflecting that the SAM binding site is also affected by the absence of the activator dimer (RBBP5-ASH2L). As we will show in the last section associated to the MD analyses, this corresponds to one of the most important factors involved in the activation mechanism of the enzyme.

#### 3.2.4. Charge Transfer Analysis

To unveil the electronic processes taking place during the reaction, we calculated the NPA charges of each fragment of the QM region, distinguishing the environment residues constituted by Tyr44, Tyr69, Tyr128 and Tyr130 with the selected water molecule (W_Y128_); and the reaction core formed by SAM and Lys4. In general, most of the environment residues showed charges below ±0.02e and in its majority close to zero. Also, no charge modifications were suffered when passing from RS to TS and also to PS in most cases. The charge transfer process occurs as expected at the reaction core conformed by SAM and Lys4, specifically at the reaction axis conformed by the fragments containing the S-C-N atoms. The values of the charge’s evolution are listed in Tables S3 and S4 from the supporting information. At the RS both Lys4 and Met group tend to have charges close to zero, while the positive charge is concentrated at the sulfur atom from SAM. During the Met transfer, as noticed from the values at the TS, the charge deficit on the sulfur atom is partially compensated by the charge transfer from Met to the sulfur as the pair of electrons from Lys4 simultaneously attack Met, process reflected in the decrease in the charge of this residue and the increase (less positive) of the overall charge of SAM while releasing the Met group. At the PS, the concerted charge transfer process is completed showing a charge deficit distributed between the Met group and the rest of the Lys4 molecule.

**Figure 7:**
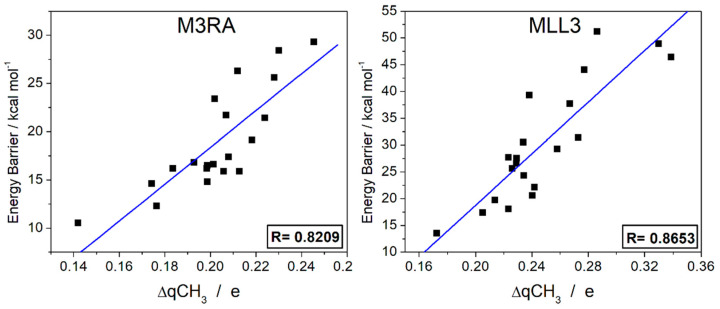
Plot of the charge transfer process taking place between the RS and the TS within the Met fragment during the methyl transfer process versus the energy barrier (∆E^≠^). The x-axis represents the change in the charge of the transferred methyl group from R to the TS.

From the partial charge analysis, we found a partial correlation between the charge transfer suffered by the Met group ΔqCH_3_) and the ∆E^≠^ as depicted in the plot from Figure 7, with correlation factors of 0.821 and 0.865 for M3RA and MLL3, respectively. If we consider that the Met group is donating a pair of electrons to the sulfur atom, while at the same time is receiving another pair from Lys4, a larger variation on the ΔqCH_3_ indicates the asynchrony of the process, where the Met group losses charge faster than it is receiving it. This result exposes the asynchrony as a factor involved in the destabilization of the Met group during the methyl-transfer process and, thus, the increase of the ∆E^≠^. Therefore, the catalytic decrease of the ∆E^≠^ is conditioned by the lowering of the positively charged character of the Met group during the reaction process. From this, it becomes clear the reasoning behind the need for proper C···N distances at the beginning of the reaction as this allow to properly start the charge transfer from Lys4 to the Met group. Also, it remarks the relevance of the oxy-anionic ring. Figure 7 shows that MLL3 presents higher variations in the Met charge during the methyl-transfer process, which as exposed above, is responsible for the increase of the ∆E^≠^.

#### 3.2.5. Product Stabilization and Specificity

The analysis of the product of the methyl-transfer was performed only for the M3RA systems, as MLL3 is not expected to reach this stage in most cases because of the high ΔE^≠^. In addition, from the comparison of the reaction energies listed in Table 2 and Table 3 for M3RA and MLL3, respectively, it is clear that the product of the methyl-transfer reaction is less stable for MLL3 than for M3RA. The reaction energies (ΔE_2_°) listed in Table 2 show an exothermic behavior with an arithmetic mean of −10.7 kcal/mol. In general, an increase in the ΔE^≠^ is related to the decrease in the exothermic behavior leading to less stable products of the methyl-transfer reaction. It has been stablished that the topology of the catalytic site with the product of the first methylation determines the feasibility towards di-methylation and probably according to this it could provide insights into the subsequent tri-methylation process. In this sense, Trievel et al. [46] suggested that the formation of hydrogen bonds between the Met group and the tyrosines of the catalytic site may determine the specificity of the enzyme. The analysis of the main interaction distances listed in Table S5 shows that there are not short-range interactions between the tyrosines and the methyl group able to justify its specificity towards monomethylation. However, the close interaction between Tyr44 and Tyr128 with Lys4 orientate the two remaining protons away from the water channel, making unfeasible their removal by a water molecule. In addition, the methyl group blocks the water channel making even more difficult the access of water molecules to the monomethylated Lys4. The only possibility would be to relocate the Met group by rotating the (Lys4)Cε-Nε to exchange its position with one of the protons from Lys4; however, this is unlikely because the presence of Tyr44 and Tyr128 which generates a steric impediment for the Met group to locate at any position besides the one that occupies after the first methyl-transfer reaction. From these results, it can be suggested that the selectivity towards mono-methylation is determined by the presence of Tyr44 and Tyr128; which could be tested by mutation of one or both residues with smaller residues providing space to locate the Met group.

### 3.3. Activation Mechanism by the Dimer RBBP5-ASH2L Binding

To identify the structural aspects involved in the activation mechanism provided by the binding of the RBBP5-ASH2L dimer to MLL3, we analyzed the differences observed from the MD simulations between M3RA and MLL3, for which we defined the main interactions between these subunits and how these could be involved in the main findings presented at this point.

**Figure 8:**
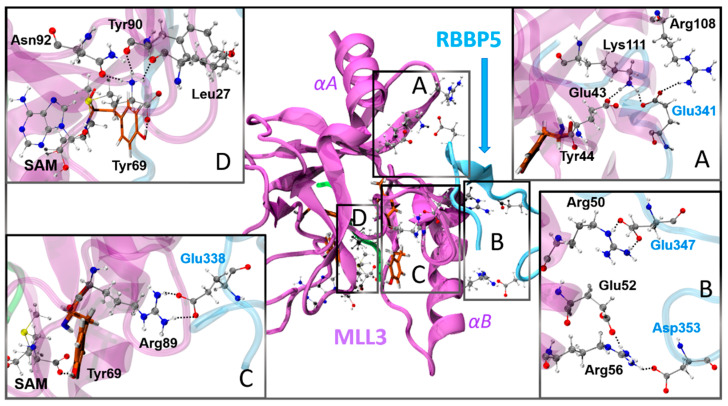
Graphical representation of the main salt-bridge interactions between MLL3 and RBBP5. There are four regions of the enzyme identified as possibly involved in the activation mechanism of MLL3 enzyme and these are enclosed in boxes (**A**–**D**). The coloring of the tertiary structure of MLL3 and RBBP5 are purple and light-blue, respectively (atom coloring: tyrosine’s carbon-orange; rest of the carbon’s―gray; red―oxygen; blue―nitrogen; white―hydrogen).

**Figure 9:**
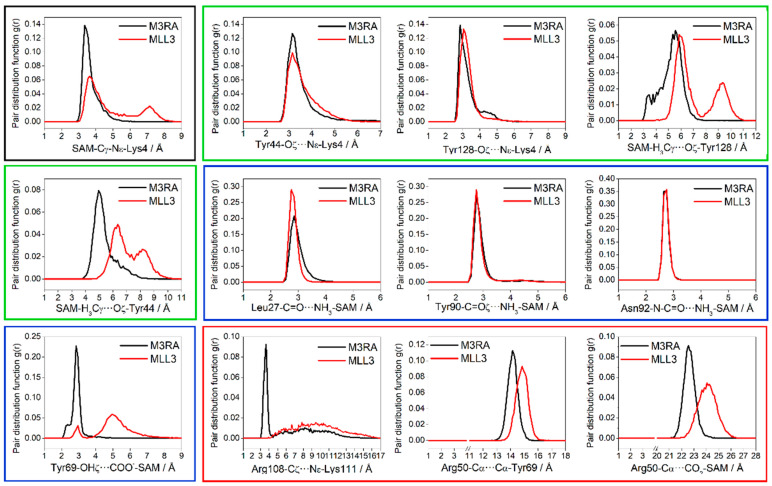
Plots of the pair distribution functions for selected distances associated to the activation mechanism sampled along 50 ns of molecular dynamic simulations of M3RA and MLL3. In the green box are the interactions involving Tyr44 and Tyr128, in the blue box are the interaction of SAM with the residues from its binding site, and in the red box are selected distances that pinpoint structural changes caused by the dimer binding to MLL3.

The MLL3-ASH2L interaction interface is dominated by both hydrophobic and hydrogen bond interactions, together with one electrostatic salt-bridge between Glu102^MLL3^···Lys137^ASH2L^ that act as the anchoring point for the complexation between these subunits. This salt-bridge is located far from the residues from the catalytic site, although it is in the vicinity of the substrate binding site so it may have a role in the stabilization of that site. Due to the large size of ASH2L, it seems to work by stabilizing the structure and positioning the small RBBP5 subunit to properly interact with MLL3, assisting the MLL3-RBBP5 binding process. On the other hand, RBBP5 interacts with MLL3 through several salt-bridges, which could restrict the conformational flexibility of MLL3, found in the proximity of the catalytic residues. Therefore, we focus our analysis of the activation mechanism on this subunit. To explore the role of these interactions we first divided the enzyme into four regions, as shown in Figure 8. In region (A) there is an interaction network that traces to Tyr44, a residue that has been found to be relevant for catalysis. This network included the pairs formed by Glu43^MLL3^···Lys111^MLL3^, Lys111^MLL3^···Glu341^RBBP5^ and Arg108^MLL3^···Glu341^RBBP5^, where in absence of Glu341^RBBP5^, Arg108 and Lys111, two positively charged residues, would repel electrostatically. Region (B) mainly involves two salt-bridges formed between Arg50^MLL3^···Glu347^RBBP5^ and Arg56^MLL3^···Asp353^RBBP5^, which accompanied by the salt-bridge from region (C), formed by Arg89^MLL3^···Glu338^RBBP5^, may have a role in restraining the *α-helixB* (*αB*) secondary structure towards the SAM cofactor binding site. Region (D) contains the cofactor binding site and its structural components may be affected by the binding of the dimer. In this region there are two main interaction points, the residues that anchor the amine group from SAM cofactor, namely Leu27, Tyr90 and Asn92; and the Tyr69 residue that anchor the carboxylic acid moiety from SAM. To provide a picture of the interaction distances distribution along the molecular dynamic simulations of the models with the deprotonated Lys4, the pair distribution functions of the main interaction distances were obtained and presented in Figure 9; while the average distances with their standard deviations are listed in Table S6 of the Supporting Information.

The selection of the deprotonated model for the analysis was because of the conformational instability observed in the simulations with the protonated Lys4-H^+^ due to the electrostatic repulsion with the SAM cofactor. This may lead towards artificial conformations, which also suggests that this state is only a transient state. Despite of that, as it will be described below, we extrapolated some observations that could be useful for understanding the role of the binding of the dimer on the deprotonation mechanism. The observed distribution of the interaction distances between the Met group and Nε-Lys4 are in line with what was proposed above from the QM/MM calculations, where MLL3 tends to present larger distances with lower population at the catalytically active distances when compared to M3RA. The interaction distances between Tyr44 and Tyr128 with Lys4 were measured from the oxygen of the hydroxyl group from the tyrosines to the Nε-Lys4 atom. In this regard, although the results of the MD simulations show only minor differences between M3RA and MLL3, it is interesting to note that the Tyr44-Oζ···Nε-Lys4 distribution is narrower and presents a higher peak in the case of M3RA, the active form. This change can be attributed to the formation of the interaction between Glu341^RBBP5^ with Arg108 and Lys111 upon formation of the trimer. These new salt-bridges stabilize the conformation of these two residues, as confirmed by the distribution of Arg108-Lys111 distances showed in Figure 9. This stabilization may in turn impact the positioning of Tyr44 because of the Lys111-Glu43 interaction (see region (A) in Figure 8).

An interesting feature raises from the observation of the distances from tyrosines 44 and 128 to the Cγ-Met from SAM: in absence of the dimer there is an increase in both the average distances and the width of the distribution. This shows that the major change along the simulation due to the formation of the active trimer was related with the proper positioning of the SAM cofactor. In this context, despite that the interactions between the amine group from SAM and the cofactor binding site were almost unaffected by the binding of the dimer, the interaction of Tyr69 with the carboxylate group from SAM was not formed along most of the simulation of MLL3, while the results of the M3RA simulation show a narrow distribution clearly peaked at hydrogen bond distances (see Figure 9). These results reveal that from a local point of view the activation mechanism is mainly modulated by the stabilization of the SAM-COO···HO-Tyr69. To shed light into the global mechanism, it is necessary to identify what changes takes places as a consequence of the formation of the salt-bridges between MLL3-RBBP5 and how these can affect the SAM-COO···HO-Tyr69 interaction. We monitored the distances between Arg50-Cα···Cα-Tyr69 and Arg50-Cα···COO-SAM that reflect the compaction of the SAM binding site upon formation of the active trimer (see regions (B) and (C) in Figure 8). As expected, the distances from the backbone from Arg50 to Tyr69 and to SAM are both shortened and narrowed in M3RA when compared with MLL3.

The fact that the main changes after the binding of the dimer involve the stabilization of Tyr69 positioning may suggest that this also involves the modulation in the conformation of the neighbor residue Val68, which was identified as essential for the deprotonation process because it assists the interaction between the deprotonating water molecule and Lys4-H^+^. Remarkably, from the data listed in Table S6, it is possible to appreciate that the interaction distances between the oxy-anion ring residues and the Met group are shorter when the system is activated, with the exception of the Val68-Met distance. We suggest that the interaction of RBBP5 with MLL3 in the active trimer restrains the residues that form the oxy-anion ring to a better suited conformation to lead the progress of the reaction, for both the deprotonation and the methyl-transfer steps.

## 4. Conclusions

In this work we have used a combination of classical MD simulations and hybrid QM/MM calculations to elucidate the activation and reaction mechanisms of mixed lineage leukemia 3 or MLL3, an enzyme in charge of the writing of an epigenetic mark through the methylation of the N-terminal domain of histone 3. The reaction consists of two steps, the deprotonation and the methyl transfer. We found the deprotonation process of the substrate lysine to be driven by a water channel with access to the catalytic site. This deprotonation step was found to be partially rate-limiting. In the methyl transfer to lysine, we found a linear correlation between the barrier height and the Met-Cγ···εN-Lys4 distance at the RS, showing that this is one of the structural parameters that control the activation process. During the methyl-transfer reaction, we found that an oxy-anion ring structural moiety stabilizes the methyl group along the transfer, predominantly controlled by dispersions interactions.

The activation mechanism caused by the formation of the MLL3-ASH2L-RBBP5 trimer (or M3RA) impacts both steps of the catalyzed reaction. This activation increases the accessibility to active conformations, whose main feature is to lead to shorter (SAM)Cγ···Nε(Lys4) interaction distances. The structural pathway to achieve those conformations is the formation of a set of salt bridges between the RBBP5 subunit and MLL3, which restrict the conformational space of specific residues of the enzyme. Such restrictions are focused on two regions: (i) an interaction network that includes Tyr44 from the catalytic site; (ii) the restriction of the distance between *α-helixB* and the SAM binding site. The latter has the largest impact in the activation mechanism. On one side, it is in charge of the apropriate positioning of Tyr69, a residue that interacts with the carboxylate group of SAM, providing an anchor for the Met group from SAM close to the Nε atom of the substrate. On the other side, the conformational restriction induced by RBPP5 optimizes the oxy-anion ring formed around the Met group of SAM, shortening the distance between the residues of the oxy-anion ring and the Met group. Formation of the trimer ensures an adequate conformation for the residues forming the ring, together with Tyr44 and Tyr69, lowering in this way the reaction barrier for the methyl-transfer step. One of the residues involved in the oxy-anion ring, Val68, was also found to assist the deprotonation of the substrate keeping a water molecule at the right position close to Lys4-H^+^.

The new insights described in our work increase our understanding of the reaction mechanism behind this epigenetic regulator, and more importantly, of the activation mechanism of MLL3, which can be crucial for the design of molecules able to modulate the activity of MLL3 and of similar enzymes.

## Figures and Tables

**Table 1:** Barrier heights (ΔE^≠^), reaction energies (ΔE_1_°) and selected geometric parameters found at the reactants and Transition Structure (TS) that reveal the main differences for the deprotonation process between M3RA and MLL3 (Distances are in Å and energies in kcal/mol). T(M)h-conf-N indicates the trimer (or monomer) configuration, where N denotes the sampling window from which it was taken.

Systems	ΔE^≠^	ΔE_1_°	Tyr44-Lys4 ^a^		Tyr128-Lys4 ^a^		Val68-W1 ^b^		Tyr69-SAM ^c^	
TRIMER			R	TS	R	TS	R	TS	R	TS
Th-conf-1	16.2	8.7	2.04	2.55	1.84	2.14	2.61	2.71	3.36	3.35
Th-conf-2	16.5	11.7	4.66	4.77	4.18	4.30	1.68	1.75	3.28	3.27
Th-conf-3	10.9	6.6	2.06	3.04	1.87	2.29	1.61	1.61	3.71	3.71
Th-conf-4	14.5	3.9	3.98	2.44	2.45	2.69	2.93	3.21	3.65	3.59
Avg. ($\bar{A}$)	14.5	7.7	3.19	3.45	2.59	2.86	2.63	2.58	3.50	3.48
MONOMER										
Mh-conf-1	33.5	10.5	5.61	5.41	4.99	4.94	3.40	3.30	5.89	5.83
Mh-conf-2	19.5	17.8	4.93	5.05	4.27	4.65	1.72	1.70	3.71	3.68
Mh-conf-3	32.5	26.8	3.65	3.29	3.07	2.99	3.14	3.05	3.82	3.59
Mh-conf-4	27.5	18.9	2.57	3.16	3.60	3.37	3.24	2.81	4.68	4.47
Avg. ($\bar{A}$)	28.3	18.5	4.19	4.23	3.98	3.99	3.22	3.13	4.53	4.39

^a^ Distance defined between the hydroxyl oxygen from Tyr and the closest proton from Lys4-H^+^. ^b^ Distance defined between the carboxyl oxygen from Val68 backbone and the closest proton from W_1_. ^c^ Interaction defined between the hydroxyl group from Tyr69 and the carboxylic acid from SAM.

**Table 2:** Barrier heights (ΔE^≠^), reaction energies (ΔE_1_°) and selected geometric parameters for the conformations at the RS and TS for the methyl-transfer reaction carried out by M3RA. The C atom refers to the carbon from the methyl group of SAM and N to the nitrogen from Lys4 side chain. Energies are in kcal/mol, distances in Å and angles in degrees (°). T-conf-N indicates the trimer configuration, where N denotes the sampling window from which it was taken.

Systems	ΔE^≠^	ΔE_2_°	C-N(RS)	C-N(TS)	S-C(RS)	S-C(TS)	S-C-N(RS)	S-C-N(TS)
T-conf-1	21.4	−11.2	3.28	2.23	1.81	2.35	143.8	171.9
T-conf-2	10.5	−18.9	2.76	2.19	1.82	2.19	163.6	170.3
T-conf-3	16.2	−12.5	2.94	2.15	1.82	2.29	162.0	167.9
T-conf-4	14.8	−20.1	3.14	2.28	1.82	2.29	153.8	174.0
T-conf-5	16.5	−16.6	3.18	2.27	1.81	2.30	148.2	172.8
T-conf-6	15.9	−17.9	3.13	2.28	1.82	2.30	147.3	173.8
T-conf-7	16.6	−12.8	3.08	2.23	1.82	2.31	159.2	175.0
T-conf-8	12.3	−21.1	2.99	2.25	1.82	2.25	156.9	173.2
T-conf-9	16.2	−14.4	3.55	2.21	1.81	2.26	158.8	167.9
T-conf-10	15.9	−14.7	3.06	2.23	1.81	2.28	133.1	168.1
T-conf-11	16.8	−13.2	3.07	2.21	1.81	2.29	153.6	172.0
T-conf-12	14.6	−10.3	2.88	2.16	1.82	2.27	170.9	175.7
T-conf-13	25.6	−2.9	3.51	2.21	1.81	2.30	146.0	174.4
T-conf-14	17.4	−16.1	3.19	2.28	1.82	2.31	152.0	172.4
T-conf-15	29.3	7.0	3.51	2.13	1.81	2.35	168.5	176.9
T-conf-16	23.4	−9.0	3.12	2.15	1.81	2.30	121.0	158.2
T-conf-17	28.4	−1.7	3.50	2.16	1.81	2.29	108.2	163.5
T-conf-18	26.3	0.1	3.54	2.17	1.82	2.32	148.4	168.7
T-conf-19	19.1	−3.4	3.25	2.18	1.82	2.36	169.4	174.2
T-conf-20	21.7	−3.5	3.19	2.12	1.81	2.35	156.0	167.2
Avg. ($\bar{A}$)	18.9	−10.7	3.19	2.20	1.82	2.30	151.0	170.9

**Table 3:** Barrier heights (ΔE^≠^), reaction energies (ΔE_1_°) and selected geometric parameters for the conformations at the RS and TS for the methyl-transfer reaction carried out by MLL3. The C atom refers to the carbon from the methyl group of SAM and N to the nitrogen from Lys4 side chain. Energies are in kcal/mol, distances in Å, angles in degrees (°) and energies in kcal/mol. M-conf-N indicates the monomer configuration, where N denotes the sampling window from which it was taken.

Systems	ΔE^≠^	ΔE_2_°	C-N(RS)	C-N(TS)	S-C(RS)	S-C(TS)	S-C-N(RS)	S-C-N(TS)
M-conf-1	20.6	−5.6	3.59	2.17	1.81	2.29	120.2	166.2
M-conf-2	17.4	−15.3	3.18	2.26	1.82	2.33	162.1	175.7
M-conf-3	22.1	−3.7	3.30	2.23	1.82	2.41	157.8	177.3
M-conf-4	25.6	−0.7	3.27	2.18	1.81	2.38	170.1	178.9
M-conf-5	19.7	−6.0	3.20	2.18	1.82	2.34	153.2	171.9
M-conf-6	48.9	−2.6	4.33	2.33	1.83	2.79	151.4	164.8
M-conf-7	46.4	0.5	4.26	2.30	1.82	2.73	151.8	162.6
M-conf-8	18.1	−13.1	3.40	2.28	1.83	2.36	165.9	176.0
M-conf-9	39.3	−7.3	4.16	2.38	1.81	2.24	111.0	156.5
M-conf-10	51.2	−7.2	4.09	2.21	1.81	2.49	115.8	151.8
M-conf-11	26.6	−22.8	3.49	2.33	1.81	2.31	124.5	170.8
M-conf-12	44.0	−4.8	3.71	2.21	1.81	2.41	108.4	149.2
M-conf-13	37.7	−8.5	3.66	2.07	1.81	2.44	104.8	148.9
M-conf-14	31.4	3.3	3.65	2.23	1.81	2.35	114.8	160.4
M-conf-15	27.5	−0.6	3.38	2.17	1.81	2.30	119.5	163.8
M-conf-16	29.3	−0.2	3.58	2.18	1.81	2.34	115.6	161.6
M-conf-17	24.3	−7.0	3.78	2.28	1.82	2.30	152.0	167.4
M-conf-18	27.7	0.3	3.39	2.17	1.81	2.29	114.2	166.1
M-conf-19	13.5	−19.3	3.11	2.15	1.81	2.24	160.3	170.3
M-conf-20	30.5	−3.5	3.53	2.18	1.81	2.29	116.2	165.2
Avg. ($\bar{A}$)	30.1	−6.2	3.60	2.22	1.81	2.38	134.5	165.3

**Table 4:** Mean values for some selected interaction distances between the cofactor and the substrate with the catalytic site, for both M3RA and MLL3 at their respective RS and TS. Distances are in Å.

Systems	M3RA		MLL3	
	R	TS	R	TS
*Tyr44-Lys4* ^a^	2.30	2.43	2.95	3.22
*Tyr128-Lys4* ^a^	2.55	2.48	2.28	2.54
Val68-Met ^b^	3.25	3.00	2.64	2.53
Arg89-Met ^b^	3.20	3.22	2.85	3.30
Ile91-Met ^b^	3.19	3.26	3.54	3.38
Tyr130-Met ^c^	3.11	2.93	3.60	2.78
Tyr69-SAM ^d^	1.57	1.57	3.01	2.99

^a^ Distance between the hydroxyl oxygen from Tyr and the closest proton from Lys4. ^b^ Distance between the carboxyl oxygen from the backbone of the residue and the closest hydrogen from Met. ^c^ Distance between the hydroxyl oxygen from Tyr and the closest hydrogen from Met. ^d^ Distance between the hydroxyl group from Tyr69 and the closest oxygen from the carboxylic acid of SAM.

## Data Availability

Not applicable.

## Supplementary Materials

The following are available online at https://www.mdpi.com/article/10.3390/biom11071051/s1, Section S1: Methyl transfer in the presence of the excess proton, Table S1: Correlation between the numbering used in this article for the MLL3 subunit and the ones used in the article from which the crystal structure was obtained, Table S2: List of experimental free energy barriers for the methyl-transfer reaction catalyzed by methyltransferase enzymes, Table S3: Quantification of the charge transfer process for M3RA taking place during the methyl-transfer reaction, Table S4: Quantification of the charge transfer process for MLL3 taking place during the methyl-transfer reaction, Table S5: Mean values for some selected interaction distances between the cofactor and the substrate with the catalytic site, for both M3RA and MLL3 at the PS, Table S6: Selected interaction distances obtained from 50 ns of MD simulations of the system with the deprotonated Lys4, Figure S1: Plot of the S···C···N angle versus the energy barrier (∆E^≠^) with their corresponding correlation factor, Figure S2: Plot of the S···C distance versus the energy barrier (∆E^≠^) with their corresponding correlation.
