# Supplementary material for: Exploration of the Activation Mechanism of the Epigenetic Regulator MLL3: A QM/MM Study
Source: Biomolecules. 2021 Jul 17;11(7):1051. doi: 10.3390/biom11071051 (PMC8301819; doi:10.3390/biom11071051)
Supplement: Supplementary file 1 [file biomolecules-11-01051-s001.zip › biomolecules-1269369-supplementary.pdf]

Exploration of the Activation Mechanism of the Epigenetic Regulator MLL3: A QM/MM Study

Sebastián Miranda-Rojas [1,*], Kevin Blanco-Esperguez [1], Iñaki Tuñón [2], Johannes Kästner [3] and Fernando Mendizábal [4]

[1] Departamento de Ciencias Químicas, Facultad de Ciencias Exactas, Universidad Andres Bello, República 275, 8370146 Santiago, Chile; k.blancoesperguez@uandresbello.edu
[2] Departamento de Química Física, Universidad de Valencia, 46100 Burjasot, Spain; ignacio.tunon@uv.es
[3] Institut für Theoretische Chemie, Universität Stuttgart, Pfaffenwaldring 55, 70569 Stuttgart, Germany; kaestner@theochem.uni-stuttgart.de
[4] Departamento de Química, Facultad de Ciencias, Universidad de Chile, P.O. Box 653, Las Palmeras 3425, Ñuñoa, 7800003 Santiago, Chile; hagua@uchile.cl
* Correspondence: sebastian.miranda@unab.cl; Tel.: +56-226618341

1. Section S1...2
2. Table S1...3
3. Table S2...3
4. Table S3...4
5. Table S4...5
6. Table S5...5
7. Table S6...6
8. Figure S1...7
9. Figure S2...8
10. References...8

Section S1. Methyl transfer in the presence of the excess proton

We explored the methyl-transfer process following right after the deprotonation to understand the timing between both stages of the reaction. For this, we used the product of the deprotonation step, meaning the Lys4 in its deprotonated form, with the proton being still part of the system as a hydronium molecule close to the exit of the water channel. The selection of the conformations was done considering the lowest and highest relative reaction energies ($\Delta E_1°$) for the deprotonation process for M3RA and MLL3. The $\Delta E_1°$ give an account of the stability of the

intermediate, therefore by considering the lowest and highest values means selecting the most stable and unstable intermediates as two extreme cases of study, in this case being Th-conf-4 and Th-conf-2 for M3RA; and Mh-conf1 with Mh-conf3 for MLL3. The resulting TSs for the methyl transfer provided $\Delta E^{\neq}$ of 47.0 and 34.9 kcal/mol for Th-conf-4 and Th-conf-2, respectively. Meanwhile, for MLL3, we obtained $\Delta E^{\neq}$ of 30.3 and 34.5 kcal/mol for Mh-conf1 and Mh-conf3, respectively. In general, these results show the impossibility of carrying out the methyl transfer without completely removing the proton from the water channel as the electrostatic repulsion between the SAM cofactor and the hydronium ion is still present. For example, in Th-conf-4 the distance between the methyl group and the hydronium ion at the RS is of 5.70Å, and even at that distance the repulsion led to an $\Delta E^{\neq}$ of 47.0 kcal/mol for the methyl-transfer. This also supports the need for the involvement of several water molecules simultaneously for the proton removal as exposed above. As we will see in the next section, in the absence of the hydronium ion, the $\Delta E^{\neq}$ is significantly reduced. These results also rule out the possibility of a deprotonation concerted with the methyl transfer step.

Table S1. Correlation between the numbering used in this article for the MLL3 subunit and the ones used in the article from which the crystal structure was obtained published by *Li et al.*[1]

Crystal structure article	*This article*
Tyr4800	Tyr44
Tyr4825	Tyr69
Tyr4884	Tyr128
Tyr4886	Tyr130

Table S2. List of experimental free energy barriers for the methyl-transfer reaction catalyzed by methyltransferase enzymes. Energy values are in kcal/mol.

Organism	Domain type	*ΔG≠ (298K)*
Human	MLL3[a]	21.3
Human	SET9[b]	20.7
Rubisco	PeaLSMT[b]	19.4
Human	RCC1-12[c]	20.2
Human	AdoMet[c]	20.2
S. pombe	CLR4[d]	18.5
Human	SET7/9[d]	20.4
Mouse	G9A	19.3
Drosophila	SU(VAR)3-9[d]	18.5
Plant homologe	pLSMT[d]	19.1
Human	Quinoline-SAM[e]	20.1

[a] reference [1]
[b] reference [2]
[c] reference [3]
[d] reference [4]
[e] reference [5]

Table S3. Quantification of the charge transfer process for M3RA taking place during the methyl-transfer reaction. Values are obtained as the charge difference between the TS and RS.

Systems	Lys4 $\Delta q_{(TS-R)}$	SAM $\Delta q_{(TS-R)}$[a]	CH_3 $\Delta q_{(TS-R)}$
T-conf-1	0.25	-0.26	0.22
Tconf-2	0.21	-0.22	0.14
T-conf-3	0.26	-0.26	0.18
T-conf-4	0.22	-0.23	0.20
T-conf-5	0.22	-0.23	0.20
T-conf-6	0.22	-0.23	0.21
T-conf-7	0.24	-0.24	0.20
T-conf-8	0.22	-0.23	0.18
T-conf-9	0.18	-0.25	0.20
T-conf-10	0.21	-0.22	0.21
T-conf-11	0.24	-0.25	0.19
T-conf-12	0.25	-0.25	0.17
T-conf-13	0.15	-0.25	0.23
T-conf-14	0.22	-0.23	0.21
T-conf-15	0.19	-0.28	0.25
T-conf-16	0.25	-0.26	0.20
T-conf-17	0.27	-0.27	0.23
T-conf-18	0.19	-0.25	0.21
T-conf-19	0.25	-0.25	0.22
T-conf-20	0.27	-0.25	0.21
Avg. (Ā)	0.23	-0.24	0.20

[a]These charge differences are calculated including the Met as part of the fragment. The remaining charge on the cofactor without the Met group can be calculated from the difference between SAM $\Delta q_{(TS-R)}$ and CH_3 $\Delta q_{(TS-R)}$

Table S4. Quantification of the charge transfer process for MLL3 taking place during the methyl-transfer reaction. Values are obtained as the charge difference between the TS and RS.

Systems	Lys4 $\Delta q_{(TS-R)}$	SAM $\Delta q_{(TS-R)}$[a]	CH_3 $\Delta q_{(TS-R)}$
M-conf-1	0.21	-0.26	0.24
M-conf-2	0.23	-0.24	0.21
M-conf-3	0.24	-0.22	0.24
M-conf-4	0.27	-0.27	0.23
M-conf-5	0.25	-0.26	0.21
M-conf-6	0.19	-0.28	0.33
M-conf-7	0.20	-0.28	0.34
M-conf-8	0.23	-0.24	0.22
M-conf-9	0.13	-0.20	0.24
M-conf-10	0.19	-0.25	0.29
M-conf-11	0.21	-0.20	0.23
M-conf-12	0.20	-0.24	0.28
M-conf-13	0.21	-0.30	0.27
M-conf-14	0.18	-0.25	0.27
M-conf-15	0.19	-0.26	0.23
M-conf-16	0.20	-0.26	0.26
M-conf-17	0.13	-0.23	0.23
M-conf-18	0.20	-0.26	0.22
M-conf-19	0.21	-0.23	0.17
M-conf-20	0.19	-0.22	0.23
Avg. (Ā)	0.20	-0.25	0.25

[a]These charge differences are calculated including the Met as part of the fragment. The remaining charge on the cofactor without the Met group can be calculated from the difference between SAM $\Delta q_{(TS-R)}$ and CH_3 $\Delta q_{(TS-R)}$

Table S5. Mean values for some selected interaction distances between the cofactor and the substrate with the catalytic site, for both M3RA and MLL3 at the PS. Distances are in Å.

Systems	*M3RA*
	PS
Tyr44-Lys4[a]	2.08
Tyr128-Lys4[a]	2.11
Val68-Met[b]	2.92
Arg89-Met[b]	2.97
Ile91-Met[b]	2.03
Tyr130-Met[c]	3.04
Tyr69-SAM[d]	1.57

[a]Distance between the hydroxyl oxygen from Tyr and the closest proton from Lys4
[b]Distance between the carboxyl oxygen from the backbone of the residue and the closest hydrogen from Met
[c]Distance between the hydroxyl oxygen from Tyr and the closest hydrogen from Met
[d]Distance between the hydroxyl group from Tyr69 and the closest oxygen from the carboxylic acid of SAM.

Table S6. Selected interaction distances obtained from 50 ns of MD simulations of the system with the deprotonated Lys4. All distances and their respective standard deviations are in Å.

Systems	*M3RA*	*MLL3*
SAM-Cγ-Nε-Lys4	3.80 ±0.52	5.70 ±1.53
SAM-Sγ-Cγ-Nε-Lys	117.1 ±22.5°	72.9 ±33.3°
Tyr44-Oζ-Nε-Lys4	3.83 ±1.01	3.83±0.72
Tyr44-Oζ-Cγ-SAM	5.60 ±0.90	7.42 ±1.11
Tyr128-Oζ-Nε-Lys4	3.51 ±0.73	3.56 ±0.73
Tyr128-Oζ-Cγ-SAM	5.48 ±0.81	7.88 ±1.67
Leu27-H$_3$N-Cγ-Met	3.04 ±0.33	2.85 ±0.16
Tyr90-H$_3$N-Cγ-Met	3.11 ±0.64	3.09 ±0.63
Ans92-H$_3$N-Cγ-Met	2.74 ±0.12	2.74 ±0.11
Tyr69-OHζ-OOC-SAM[a]	2.17 ±0.73	4.85 ±1.31
Val68-Met[a]	4.39 ±1.34	3.67 ±0.54
Arg89-Met[a]	3.67 ±0.62	5.04 ±1.13
Ile91-Met[a]	4.14 ±0.61	6.66 ±1.20
Tyr130-Met[c]	3.92 ±0.44	5.67 ±1.04
Arg50-Tyr69[d]	14.1 ±0.37	14.9 ±0.42
Arg50-SAM[e]	22.5 ±0.48	24.1 ±0.74
Glu43-Lys111[f]	3.60 ±0.36	4.18 ±1.24
Arg108-Lys111[g]	9.89 ±2.52	11.09 ±2.65
Arg89-Arg108[h]	24.35 ±2.96	25.43 ±2.89

[a]Distance between the hydroxyl group from Tyr69 and the closest oxygen from the carboxylic acid of SAM.
[b]Distance between the carboxyl oxygen from the backbone of the residue and the *C*γ from the Met group from SAM.
[c]Distance between the hydroxyl oxygen from Tyr and the *C*γ from the Met group from SAM.
[d]Distance between the Cα from the backbone of both residues.
[e]Distance between the Cα from the backbone of Arg50 and the carbon from the carboxylate group of SAM.
[f]Distance between the carbon from carboxylate group of the side chain of Glu43 and the nitrogen from the side chain of Lys111.
[g]Distance between the carbon from guanidinium group of the side chain of Arg108 and the nitrogen from the side chain of Lys111.
[h]Distance between the carbons from guanidinium groups of the side chain of Arg89 and Arg108.

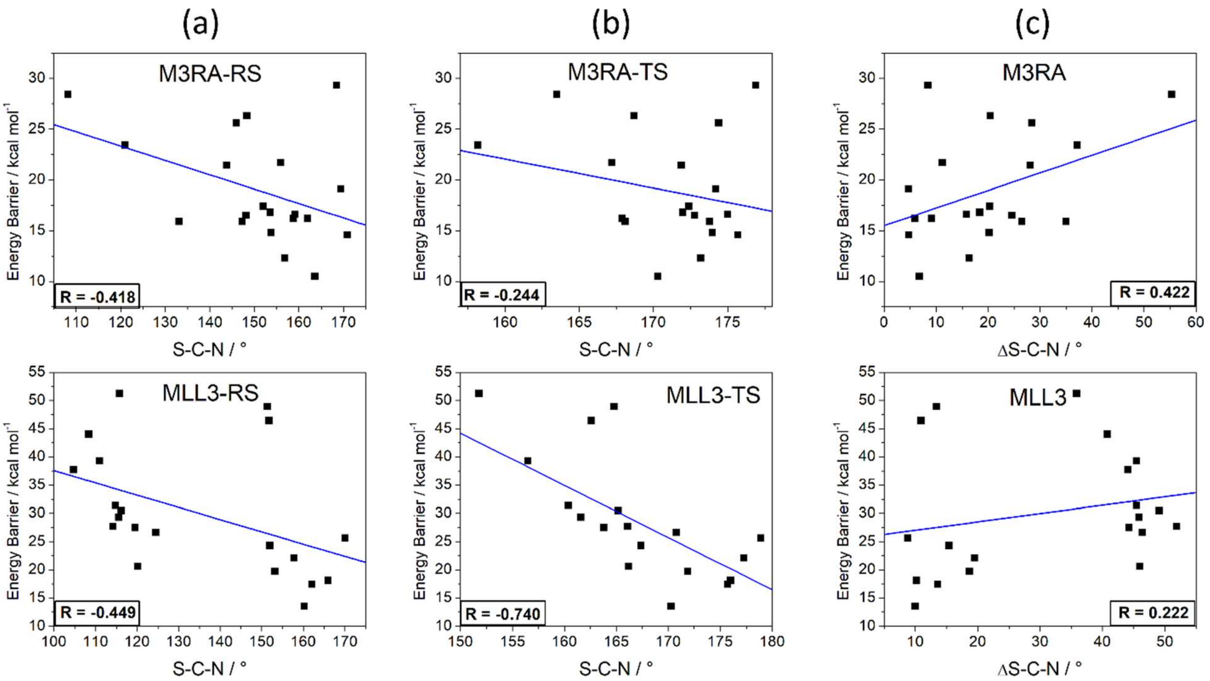

Figure S1 Plot of the S···C···N angle versus the energy barrier (ΔE≠) with their corresponding correlation factor (a) Reactant state (b) Transition state (c) Difference between reactant state and transition state

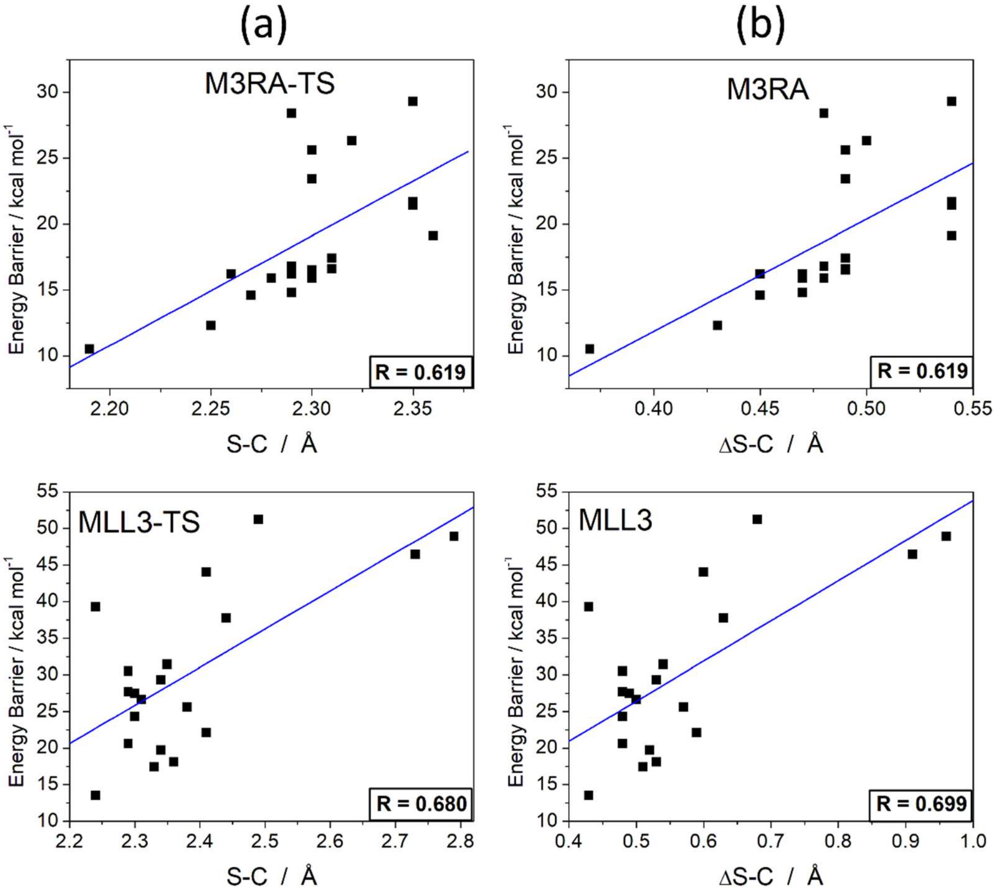

Figure S2. Plot of the S···C distance versus the energy barrier (ΔE≠) with their corresponding correlation factor (a) Transition state (c) Difference between reactant state and transition state. The Reactant state was omitted because they variated within a very short range going from 1.81 to 1.83 Å.

References

1 Y. Li, J. Han, Y. Zhang, F. Cao, Z. Liu, S. Li, J. Wu, C. Hu, Y. Wang, J. Shuai, J. Chen, L. Cao, D. Li, P. Shi, C. Tian, J. Zhang, Y. Dou, G. Li, Y. Chen and M. Lei, *Nature*, 2016, **530**, 447–452.

2 D. Patnaik, H. G. Chin, P.-O. Estève, J. Benner, S. E. Jacobsen and S. Pradhan, *J. Biol. Chem.*, 2004, **279**, 53248–53258.

3 S. L. Richardson, Y. Mao, G. Zhang, P. Hanjra, D. L. Peterson and R. Huang, *J. Biol. Chem.*, 2015, **290**, 11601–11610.

4 E. Collazo, J.-F. Couture, S. Bulfer and R. C. Trievel, *Anal. Biochem.*, 2005, **342**, 86–92.

5 H. S. Loring and P. R. Thompson, *Biochemistry*, 2018, **57**, 5524–5532.
